# Caloric Restriction May Help Delay the Onset of Frailty and Support Frailty Management

**DOI:** 10.3389/fnut.2021.731356

**Published:** 2021-09-06

**Authors:** Pan Liu, Yun Li, Lina Ma

**Affiliations:** ^1^Department of Geriatrics, Xuanwu Hospital, Capital Medical University, Beijing, China; ^2^National Clinical Research Center for Geriatric Medicine, Beijing, China

**Keywords:** caloric restriction, frailty, older adults, sarcopenia, lifespan

## Abstract

Frailty is an age-related clinical syndrome that may increase the risk of falls, disability, hospitalization, and death in older adults. Delaying the progression of frailty helps improve the quality of life in older adults. Caloric restriction (CR) may extend lifespan and reduce the risk of age-related diseases. However, few studies have explored the relationship between CR and frailty. In this review, we focused on the impact of CR on frailty and aimed to identify potential associated mechanisms. Although CR may help prevent frailty, further studies are required to determine the underlying mechanisms and specific CR regimens suitable for use in humans.

## Highlights

- Caloric restriction (CR) has antiaging effects and great significance in delaying frailty and sarcopenia.
- Some suggestions about CR for frailty are proposed.
- Further study is needed to determine the mechanisms and detailed CR interventions appropriate for humans.

## Introduction

Frailty is an age-related clinical geriatric syndrome associated with the decline of multiple physiological systems and increased risk of adverse health outcomes, such as falls, hospitalization, disability, and premature mortality in older adults (1, 2). Frailty is receiving increasing research and clinical attention due to the rapid population aging. The prevalence of frailty is estimated in the range of 4–59% (3). The Fried phenotype (1) and the frailty index (FI) (4) are two widely used methods for frailty assessment. The frailty phenotype includes features, such as unintentional weight loss, poor muscle strength, exhaustion, reduced physical activity, and slow walking speed (1). Interventions that prevent or delay the onset of frailty are required to improve the quality of life among older adults. The previous studies have examined frailty in rodents (5–8) and humans (9).

High-calorie diets are risk factors for obesity and metabolic diseases (10). Caloric restriction (CR) is defined as a reduction in energy intake (typically by 20–40% of *ad libitum* consumption) without malnutrition (11). CR has been reported to considerably extend a healthy lifespan and prevent age-related diseases in both animals and humans (11–14). However, the previous studies have mainly focused on the association between CR and aging, and few studies have explored the relationship between CR and frailty. This review aimed to summarize the evidence on the impact of CR on frailty and to explore candidate underlying mechanisms.

## CR and Frailty

In the clinical setting, the FI may be a lifespan biomarker, helping in predicting age-related mortality (15). A previous study has shown that 30% CR may enhance strength in both old- and middle-aged male mice and improve balance and motor coordination in both old- and middle-aged female mice; these outcomes are closely associated with a delay in the onset of age-related frailty (16). In addition, a separate study has shown that both middle-aged and old male mice with the CR of 30% had grip strength greater than that observed in their counterparts (17). Old male C57BL/6 mice that consumed a 40% CR diet over 13 months period, starting from 6 months of age, and that fed an *ad libitum* diet combined with 6 months of resveratrol treatment both improved frailty status compared with their counterparts. However, this difference was not observed in female mice (18). In contrast to the C57BL/6 mice, CR did not delay age-related decline in DBA/2 mice. Male DBA/2 mice on a similar CR diet had a higher risk of frailty than did the matched C57BL/6 mice. There was no difference in frailty assessment by FI among both sexes of CR mice (18). The impact of CR regimens on frailty, activity, and memory in male Wister rats was stratified by CR starting point and duration. A CR of 40% imposed over 6, 12, or 18 months, starting at 6 months of age, improved the general locomotor activity and spatial memory and decreased the age-related frailty. However, the benefits of CR started in late adulthood were unclear; for example, a CR of 3 months starting at the age of 15 and 21 months increased the risk of frailty in old rats (19). Most studies on CR have been conducted in male animals. Further studies in female mice and rats or other species are required.

A 4-year treatment involving 30% CR beginning in adulthood (3.2 ± 0.1 years of age) may extend lifespan by 50% and reduce the risk of age-related diseases in male gray mouse lemurs, without affecting motor and cognitive performance (20). Meanwhile, 30% CR may extend the health span in rhesus monkeys (21). In the same species, Yamada et al. have shown that long-term 30% CR started in adulthood may reduce the incidence of frailty by improving weakness, endurance, slowness, and physical activity and extend healthy lifespan in both the sexes (22).

An interleukin-10 knockout (IL-10^−/−^) mouse model is the genetic model of frailty (8). However, few studies on CR have used this model. Rapamycin, an inhibitor of mammalian target of rapamycin (mTOR), may improve muscle function and prevent frailty in IL-10^−/−^ mice (23). Cu/Zn superoxide dismutase knockout mouse (Sod1^−/−^) is another model of frailty, with characteristics similar to those observed in humans with frailty, such as weight loss, weakness, reduced physical activity, and exhaustion (7). The studies have shown that 40% CR may attenuate age-related loss of muscle mass of Sod1^−/−^ mice by improving mitochondrial function, reducing oxidative stress damage and cellular senescence, and decreasing IL-6 levels (24, 25). Upregulation of SIRT3 and mitochondrial antioxidant manganese superoxide dismutase expression in CR Sod1^−/−^ mice may help protect against muscle damage (24).

### CR and Frailty in Humans

The Comprehensive Assessment of Long-Term Effects of Reducing Intake of Energy (CALERIE) trial has shown that 6 months of 25% CR reduced the levels of fasting insulin and body temperature in overweight adults (26). Further studies have shown that extending CR for 2 years may improve chronic inflammation markers, blood pressure, the levels of glucose, and blood lipids, alongside other cardiovascular metabolic indicators in young and middle-aged healthy adults (27), while improving cognitive function in non-obese healthy adults (28). Dora et al. found that this CR regimen reduced oxidative stress in male and female adults, as indicated by the urinary concentration of F2-isoprostane (29). Another study indicated that 12 weeks of CR improved cardiometabolic health in sedentary adults with obesity and aged ≥65 years (14). Age-related loss of skeletal muscle quantity and quality is associated with reduced gait speed and overall strength and a high risk of fall and frailty. A previous study has shown that 15–25% CR may prevent age-related muscle atrophy in humans (30), potentially improving the frailty. Other studies have shown that time-restricted feeding improved the walking speed and quality of life in overweight sedentary older adults (aged ≥65 years) (31). However, the generalizability of these findings requires further research.

### CR and Sarcopenia

Sarcopenia is an age-related syndrome of muscle strength and functional decline that is closely associated with frailty; in fact, it may contribute to physical frailty. CR exerts a protective effect against sarcopenia in both rodents and non-human primates (32–35). A CR of 30% over 10 weeks may improve skeletal muscle function in male C57BL/6 mice (33). Lifelong 8% CR prevents age-related disruption of the myofiber membrane environment in male Fischer-344 rats (32). The effects of different durations (2.5, 8.5, and 18.5 months) of 40% CR on skeletal muscle may depend on animal strain, sex, and age (36). Vastus lateralis biopsies collected at 6, 9, and 12 years after the treatment that included a 30% CR diet have shown that CR may prevent the shift in fiber type distribution and delay cellular atrophy in male rhesus monkeys (34).

## Possible Mechanism Of CR Effects On Frailty

**Figure 1:**
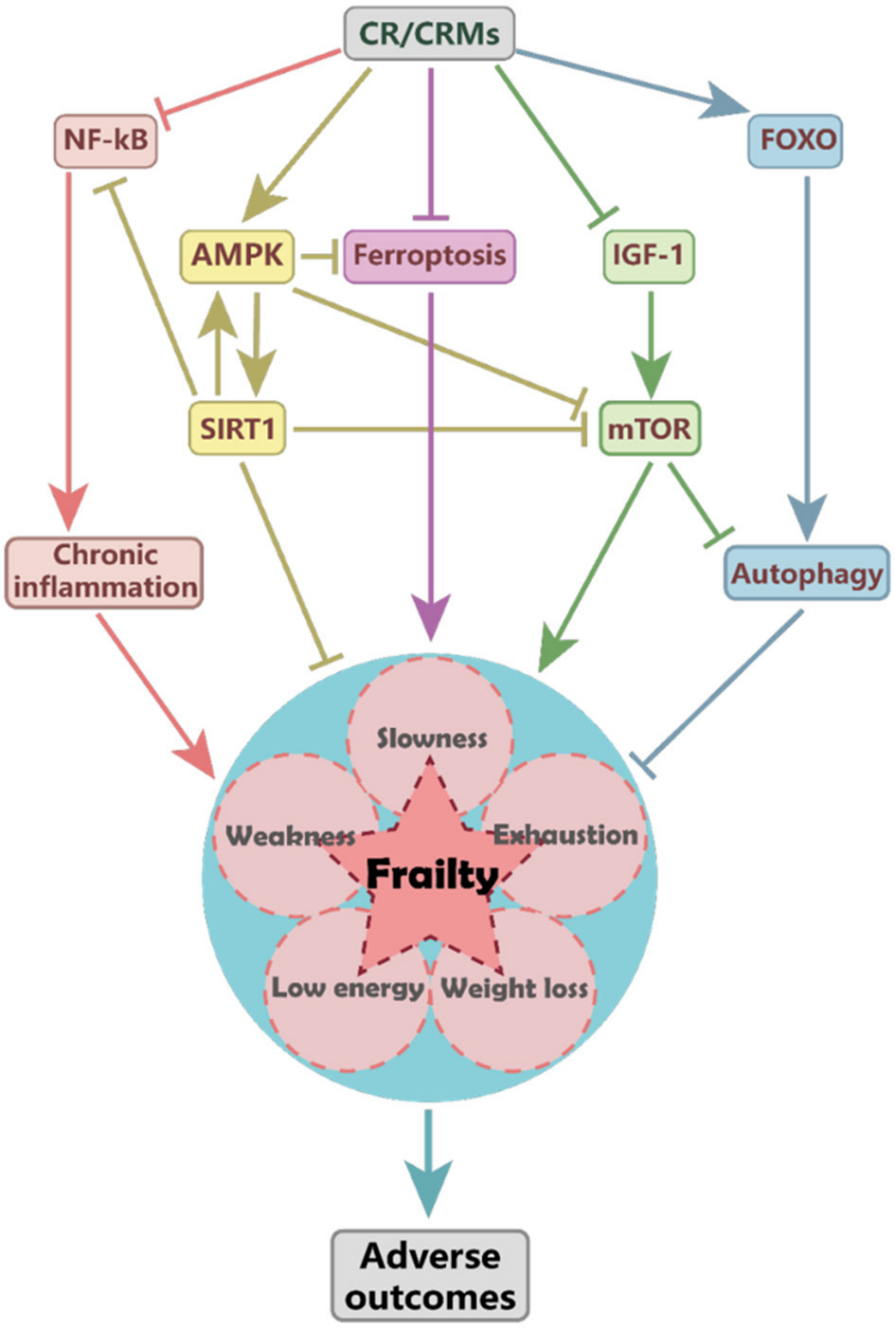
The proposed mechanism of caloric restriction (CR) impacts frailty. CR may reduce the risk of frailty and associated adverse outcomes by activating the AMPK and SIRT1 pathways, inhibiting the IGF-1 and mTOR signaling and ferroptosis, and reducing the inflammation mediated by NF-KB pathways. CR-induced SIRT1 activation may upregulate AMPK and suppress NF-κB and mTOR activity. CR and metformin may attenuate ferroptosis by activating the AMPK pathway and improving frailty. AMPK, adenosine 5′-monophosphate (AMP)-activated protein kinase; CR, caloric restriction; CRMs, caloric restriction mimetics; FOXO, forkhead box O; IGF-1, insulin-like growth factor-1; mTOR, mammalian target of rapamycin; NF-κB, nuclear factor-κB; SIRT1, silent mating-type information regulation 2 homolog 1.

The mechanisms of CR impact on frailty remain unclear; several target pathways involved in antiaging may be affected, such as the inhibition of insulin-like growth factor-1 (IGF-1) and mTOR signaling, activation of adenosine 5′-monophosphate-activated protein kinase (AMPK) and sirtuins, and promotion of autophagy (Figure 1) (12). Sirtuins are a conserved family of nicotinamide adenine dinucleotide (NAD)-dependent proteins. Silent mating-type information regulation 2 homolog 1 (SIRT1) and other sirtuins may mediate the protective effects of CR (37). SIRT1 activation may extend lifespan through the activation of AMPK, which further inhibits mTOR, promotes lipid catabolism and gluconeogenesis (38). CR may delay cognitive decline in mice by modulating the SIRT1/mTOR signaling pathway and by activating SIRT1 and suppressing mTOR signaling (39).

Lower sirtuin levels are independently associated with frailty, regardless of age, sex, and comorbidities. Lower circulating levels of SIRT1 and SIRT3 may indicate frailty (40), and frail older adults are more likely than their counterparts to have lower serum-induced SIRT1 expression levels (41). In contrast, the previous study has shown that frail older patients had higher levels of SIRT1 than did their counterparts. Older adults with elevated SIRT1 levels had decreased physical function (42). Nevertheless, serum-induced SIRT1 expression has not been associated with frailty (43). Further studies are required to elucidate the relationship between SIRT1 and frailty and other signaling pathways that may mediate the relationship between CR and frailty.

Cell senescence and chronic inflammation are important characteristics of aging and frailty (44); CR exhibits anti-senoinflammatory effects by suppressing the expression of cytokines and chemokines in the senescence-associated secretory phenotype. The CR mimetics (CRMs) may improve the dysregulated activity of signaling pathway molecules (45). A CR diet may delay the onset of frailty and improve the progression of several chronic diseases by reducing the development of chronic low-grade inflammation (46), associated with elevated levels of C-reactive protein, IL-1β, IL-6, and tumor necrosis factor-α (TNF-α) (47). Moreover, CR exhibits considerable anti-inflammatory activity by modulating the activity of nuclear factor-κB (NF-κB) and forkhead box O (FOXO) (48). Activation of SIRT1 may suppress the NF-κB pathway (49). Immune senescence is a natural consequence of aging that is associated with frailty. CR may attenuate age-related changes of the natural killer cells and T cells to preserve immune function in later life, which is a system-wide effect (50).

Iron dyshomeostasis and ferroptosis may trigger cell and organismal death in *Caenorhabditis elegans* (51). CR and metformin attenuate ferroptosis by activating the AMPK pathway, which has been associated with extended lifespan and health span and improved frailty (51). CR may protect against cognitive function decline by inducing senescence-accelerated prone eight astrocytes protective gene expression and functional rejuvenation *in vitro* (52). In addition, CR may improve insulin sensitivity (11) by mediating the adipose mTOR2 pathway; however, the activity of this pathway is not necessary for the beneficial effects of CR (53).

Age-related apoptosis in skeletal myocytes may lead to sarcopenia, which involves mitochondria- and TNF-α-mediated pathways (54). Interventions targeting myonuclear apoptosis improve sarcopenia and physical frailty symptoms (55). Lifelong 8% CR has been shown to reduce age-related rates of apoptosis and oxidative damage to the skeletal myocyte by regulating autophagy in rats (56). This mechanism may be associated with heat shock protein 27 signaling, which, when insufficient, may contribute to apoptosis and muscle wasting (57). The upregulation of the IGF1-Akt-mTOR-FOXO signaling pathway may accelerate sarcopenia in aged mice (58). CR may help preserve muscle mass in middle-aged rats by downregulating mTOR and ubiquitin-proteasome pathway signaling (59). Further, CR may delay skeletal muscle aging in rhesus monkeys by inducing metabolic changes (60). These findings indicate that CR may delay sarcopenia by reducing oxidative stress damage, inflammation, and iron overload, as well as improving mitochondrial function, enhancing protein homeostasis, and increasing autophagy and apoptosis (61).

## Types of CR

Caloric restriction has been reported to extend health span and lifespan and prevent age-related diseases and frailty. However, the optimum timing of CR initiation or duration remains unclear as few previous studies have focused specifically on frailty. Further studies are required to establish regimens most likely to improve the quality of life of older adults. At the time of writing, several types of CR regimens exist. For example, the Mediterranean CR diet has been shown to decelerate age-related cognitive decline (62) and the progression of aging and prevent frailty (63), making this approach useful for frailty management in the clinical context (64). The clinical impact of CR may depend on the factors, such as compliance; herein, we describe candidate approaches to CR that include intermittent fasting, CRMs, and protein dietary restriction.

### Intermittent Fasting

No diet regimen is suitable for everyone. Different from continuous CR, intermittent fasting consists of periods of little or no energy intake and intervening periods of normal food intake (65), which have benefits for weight loss, healthy aging, and chronic disease prevention (66), such as improving cardiometabolic health in overweight and obese individuals (67). In addition, intermittent fasting may play an important role in reducing oxidative stress, improving insulin sensitivity, repairing autophagy, and improving cognitive function (65). Established intermittent fasting regimens determined by the interval length of fasting (66, 68) include time-restricted feeding, alternate-day fasting, alternate-day modified fasting, and the 5:2 diet. For example, the 5:2 diet involved 2 days of fasting with no more than 25% energy intake and 5 days of regular eating patterns per week (67). Time-restricted feeding may help protect cardiometabolic health; in contrast to CR, it may also be associated with satisfactory compliance as time is relatively easy to monitor (69). In later life, intermittent fasting on alternate days may increase renal gasotransmitter hydrogen sulfide production, which may help reduce age-related frailty in male mice (70).

### CR Mimetics

Caloric restriction mimetics are compounds that mimic physiological and metabolic CR effects (71), such as resveratrol, rapamycin, metformin, NAD precursors, and senolytics (15). They have positive effects on the rodent lifespan and human health and are used in interventions against aging and age-related cardiovascular, neurodegenerative, and malignant diseases (72). Moreover, these compounds may help prevent age-related frailty, as assessed using the FI in mice (15). Several CRMs have been shown to prevent frailty (Table 1); for example, 6 months of resveratrol treatment (100 mg/kg/day) starting at 18 months of age has been shown to prevent frailty in mice (18). In addition, 6-week resveratrol treatment (150 mg/kg/d) has been shown to improve the grip strength and muscle mass in aged rats through the activation of the AMPK/SIRT1 pathway (73). SRT1720, another SIRT1 activator, may extend lifespan and improve the health of mice through SIRT1 activation and NF-κB expression reduction (74). Frailty is associated with SIRT1 activity in older adults (42); targeting this pathway with CRMs, such as resveratrol may affect both robustness and frailty in humans (37); Metformin has been reported to extend the lifespan of older adults with type 2 diabetes by preventing frailty (75). Exposure to any dose or frequency of metformin administration may reduce the risk of frailty in older adults (76). An 18-month intervention involving rapamycin (1.5 mg/kg/d) for IL-10^−/−^ mice has been shown to prevent muscle mass loss and frailty by decreasing myostatin levels (23). Meanwhile, 12-week treatment with low-dose oral rapamycin (0.5, 1, and 2 mg) failed to improve the frailty status in older adults with coronary artery diseases (77). The combination of dasatinib (5 mg/kg) and quercetin (50 mg/kg), as one of the senolytics, may extend health span and alleviate symptoms of frailty in aged mice (78). In addition, a chronic nicotinamide diet, an NAD+ precursor, at doses in the range of 0.5 or 1.0 g/kg, can improve the health span but not the lifespan of adult mice (79). Future studies are required to elucidate the effects of CRMs on frailty.

**Table 1:** Caloric restriction mimetics and frailty assessments.

**CRMs**	**Category**	**Species**	**Onset**	**Dose and duration**	**Frailty assessment**	**Results**
Resveratrol	SIRT1 activator	Male, C57BL/6J mice	18 months	100 mg/kg/d, 6 months	Mouse FI	Reduces FI scores (18)
		Male, SD rats	24 months	150 mg/kg/d, 6 weeks	Physical function	Improves grip strength and muscle mass (73)
SRT1720	SIRT1 activator	MaleC57BL/6J mice	7 months	100 mg/kg/dNatural death	-	Extends lifespan and improves health in mice (74)
Metformin	AMPK activator	Adults aged ≥65 years with type 2 diabetes	Receiving metformin in outpatient care	–	FI	Reduces risk of frailty regardless of dose and frequency (76)
Rapamycin	mTOR inhibitor	IL-10^−/−^ mice	6 weeks	1.5 mg/kg/d, 18 weeks	Mouse FI	Decreases levels of myostatin which may prevent muscle mass loss and frailty (23)
Dasatinib and quercetin	Senolytic drugs	Male, C57BL/6J mice	20 months 24–27 months	A combination of dasatinib (5 mg/kg) and quercetin (50 mg/kg) 4 months Natural death	Physical function	Alleviates symptoms of frailty and extends healthspan (78)
Nicotinamide	NAD+ precursor	Male, C57BL/6J mice	56 weeks	0.5 and 1.0 g/kg, 62 weeks	–	Improves healthspan but does not extend lifespan (79)

*AMPK, adenosine 5′-monophosphate-activated protein kinase; CRMs, caloric restriction mimetics; FI, frailty index; IL-10^−/−^, interleukin-10 knockout; mTOR, mammalian target of rapamycin; NAD, nicotinamide adenine dinucleotide; SD, Sprague-Dawley; SIRT1, silent mating-type information regulation 2 homolog 1*.

### Protein Diet

Macronutrient balance is important for healthy aging. Higher protein intake has been associated with worse frailty status over time in a relatively healthy population; no similar effect has been identified for either carbohydrates or fats (80). Further, low-protein high-carbohydrate diets may help expand lifespan (81). Protein restriction has been shown to affect the rodent lifespan in a manner similar to that associated with CR (81, 82). Amino acids, particularly branched-chain amino acids (BCAAs), such as leucine, isoleucine, and valine, are associated with improved health and increased lifespan in different organisms (83, 84). Protein restriction may increase the risk of frailty and sarcopenia (85). Intake of a BCAA-enriched balanced amino acid mixture may help preserve muscle fiber quantity, improve motor coordination and endurance, and extend the lifespan of middle-aged mice by modulating the mTOR/eNOS pathway, which affects mitochondrial biogenesis (84). In addition, a BCAA-enriched diet may help prevent disability and extend a healthy lifespan in older adults (86), suggesting that this diet may be suitable for older adults at risk of frailty. In contrast, Richardson et al. suggested that lifelong restriction of dietary BCAAs may extend lifespan and prevent frailty in aged male mice. Nonetheless, the effect of the BCAA diet on frailty remains unclear (87). Further studies are needed to examine these associations in humans. The controversies regarding the effects of BCAA dietary restriction or enrichment may be associated with different factors, such as intervention onset, duration, and species. Further studies are required to elucidate the relationship between protein intake, lifespan, and age-related diseases.

## Combination Of CR and Exercise

Diet and exercise are critical components of healthy aging. Protein supplementation alone may not alleviate sarcopenic symptoms (30). Protein supplementation combined with resistance training is recommended to prevent sarcopenia and frailty (64). The previous studies have shown that a combination of resistance training and CR for 6 months may improve maximal strength in menopausal women with obesity (88). Meanwhile, other studies have shown that CR combined with resistance training may prevent CR-induced muscle loss in older adults with obesity (89). A separate study has shown that the interventions involving CR and exercise may improve age-related conditions in adults with type 2 diabetes (90). Thus, exercise may be considered as another type of CRMs, helping prevent frailty and improve healthy aging alone or in combination with CR (91). These effects are likely mediated by antioxidant-related mechanisms (91). However, it should be noted that the combination of CR and aerobic exercise training practiced for 5 months may not affect cognition in sedentary older adults with obesity (92). Thus, further investigations are required to determine lifestyle interventions suitable for older adults and those with frailty or sarcopenia.

## Potential Risks Associated With CR

Malnutrition is common in older adults and increases the risk of frailty, sarcopenia, comorbidities, and premature death. CR may delay the onset of frailty and sarcopenia, potentially helping to improve the quality of life of older people. However, extreme CR may lead to adverse events, such as sarcopenia, osteoporosis, and immune deficiencies (93). Aged rats with 3 months of CR had poorer performance and frailty scores than their counterparts (19). This finding was consistent with that of another study showing that 40% CR initiated in mice aged 22–24 months increased mortality rates in male C57BL/6, DBA/2, and B6D2F1 mice (94). Further, CR accelerated the loss of gray matter but preserved the white matter in the brain of aged mouse lemurs; neither effect altered the cognitive performance (20). While chronic food restriction may impair spatial recognition memory in developing mice (an effect mediated by the extent of food restriction and individual tolerability), acute food restriction exerts negative effects on locomotor activity in mice (95). The relationships between CR, genetics, sex, animal strains, as well as regimen duration and extent, are complex. Future studies are required to elucidate the suitable timing, duration, and extent of CR that may help prevent the onset of frailty in older adults.

## Conclusion

Caloric restriction has shown some benefits in both animal and human studies; however, the factors that determine the impact of CR remain unclear (19). Rodent and non-human primate models of CR are associated with the limitations that may affect study designs. The impact of CR on aging may be mediated by dietary composition, sex, age at onset, feeding regimens, and genetics (96). There is no standard for CR regimens (e.g., timing of initiation and duration, or caloric intake values). In addition, the evidence on the association between CR and frailty in the clinical setting is insufficient. Moreover, the underlying mechanisms are unclear. Consequently, further studies are required to elucidate the caloric intake and nutrient composition optimal for healthy aging in humans.

## Author Contributions

PL, YL, and LM contributed to the organization of the manuscript. PL drafted the manuscript and composed the outline. YL and LM reviewed and approved the submitted version. All the authors agree to be accountable for the content of the study.

## Conflict of Interest

The authors declare that the research was conducted in the absence of any commercial or financial relationships that could be construed as a potential conflict of interest.

## Publisher's Note

All claims expressed in this article are solely those of the authors and do not necessarily represent those of their affiliated organizations, or those of the publisher, the editors and the reviewers. Any product that may be evaluated in this article, or claim that may be made by its manufacturer, is not guaranteed or endorsed by the publisher.
